# Commentary: The Historical Roots of Visual Analog Scale in Psychology as Revealed by Reference Publication Year Spectroscopy

**DOI:** 10.3389/fnhum.2021.711691

**Published:** 2021-07-15

**Authors:** Kenpei Shiina

**Affiliations:** Department of Educational Psychology, School of Education, Waseda University, Tokyo, Japan

**Keywords:** visual analog scale, graphic rating scale, history, origin, rating scales

Yeung and Wong's study is an example of how modern information retrieval technology can produce good results. It seems to clearly indicate that Hayes and Patterson (1921) is the origin of the visual analog scale (VAS). However, this commentary reserves the conclusion that Hayes and Patterson (1921) is the only source of the VAS, and provides a more comprehensive and proper historical perspective for future studies using rating scales.

First of all, Hayes and Patterson (1921) did not introduce the VAS directly but introduced the graphic rating scale (GRS). Since the GRS is very similar to the VAS, it seems reasonable to conclude that the VAS originated from Hayes and Patterson (1921). However, a closer examination of the context of Hayes and Patterson's paper reveals that it is an abstract of a conference held in 1920 (Boring, 1921) and is very concise and somewhat crude (for example, the second author's name has an error: the correct name is *Paterson* instead of *Patterson*). Therefore, it is difficult to obtain an overview of the GRS from this paper alone. If we seek further information, we notice that the authors belong to Scott Co. Moreover, it is easy to see that Scott is the famous Walter Dill Scott by referring to Freyd (1923) listed in Table 1 of Yeung and Wong (2019). Therefore, it can be speculated that Hayes and Patterson's paper reflects the collaborative work at Scott Co. If we examine Scott's own research, we notice that Freyd (1923) did cite Scott (1920) and a book of Scott and Hayes (1921). This book includes a description and a figure of the GRS around p. 96. The earliest paper of Scott (1920) on the GRS published as an article in a commercial magazine also contains a GRS figure.

It can be said that the GRS was introduced gradually. Scott introduced the GRS in a commercial magazine in November 1920. Hayes and Patterson made a conference presentation in December 1920 and in 1921 its abstract, which Yeung and Wong (2019) and other researchers considered the origin of the VAS, was published. Scott and Hayes published a book in 1921. An important commentary, which was not captured by Yeung and Wong's procedure, was made by Paterson in 1922, who stated that Ruml made a significant contribution. Freyd's good review article was published in 1923. Scott and Clothier's book in the same year provided a detailed explanation of the GRS. This book is famous in industrial psychology and, although there is no direct evidence, this book appears to contain the same data presented in Hayes and Patterson (1921). The GRS researchers mentioned so far, namely, Hayes, Paterson (Patterson), Ruml, Freyd, Scott, and Clothier, were members of Scott Co., with the exception of Freyd, and were all active primarily in industrial-organizational psychology. Vinchur (2018) provides more information on the careers and achievements of these researchers and the activities of Scott Co. The related figures of the classical rating scales are provided by Shiina (2021).

In conclusion, the GRS should be seen as a joint achievement of Scott Co., as clearly indicated by the title of Paterson (1922). Notice that this *Paterson* is the second author of Hayes and Patterson (1921). At the same time, the excellent results of the information retrieval technology do not appear to be perfect. Care may be needed, especially when dealing with classical books and non-academic papers. This may mean that careful deciphering of the literature is still needed for a while. Because Scott (1920) and Scott and Hayes (1921) were undercited by the academic literature (for example, there is zero detection by Web of Science database), it was challenging to discover them by cited reference analysis alone. In particular, CRExplorer used by Yeung and Wong (2019) had a limitation in conducting cited reference analysis at least in the present case, so it may be recommendable that we use a much broader (but less accurate) database such as Google Scholar in similar cases.

The GRS is not an individual achievement but a joint achievement of Scott Co. Therefore, in the author's opinion, the origin of the GRS (and the VAS) should be Scott and Hayes (1921) in terms of content and Scott (1920) in terms of time. Hayes and Patterson (1921) is probably the third most important contribution. Therefore, it seems appropriate to cite these three papers together. Furthermore, it may be reasonable to include Scott and Clothier's book in 1923, which integrates the fragmented studies cited above, or to cite this book alone (Scott and Clothier, 1923).

If one still wants to name the sole inventor, Walter Dill Scott will be it.

## Author Contributions

The author confirms being the sole contributor of this work and has approved it for publication.

## Conflict of Interest

The author declares that the research was conducted in the absence of any commercial or financial relationships that could be construed as a potential conflict of interest.
